# A rapid scoping review of antibiotic access and use barriers among refugee and migrant populations

**DOI:** 10.1186/s12992-026-01188-x

**Published:** 2026-01-29

**Authors:** Suzanne Garkay Naro, Michèle Palkovits, Arne Ruckert, Andrea Morales Caceres, Ranjana Nagi, Cordelia Chik, Steven J. Hoffman, Susan Rogers Van Katwyk

**Affiliations:** Global Strategy Lab, School of Global Health, York University, Toronto, ON M3J 1P3 Canada

**Keywords:** Antibiotic resistance, Migrants, Refugees, Global health

## Abstract

**Background:**

Antibiotic resistance (ABR) poses a significant global health and development threat from increasing globalization of travel, trade, and animal and human migration. ABR impacts refugees and migrants in unique ways due to increased exposure to infections and inequitable access to healthcare. The objective of this review is to synthesize the evidence on access to and appropriate use of antibiotics by migrants and refugees, identify the barriers they may experience in accessing and using antibiotics, and reflect on global policy entry points to modify such barriers considering the persistent globalization-related impacts on ABR.

**Methods:**

A global rapid scoping review was conducted to collect evidence on barriers to access and appropriate use of antibiotics among migrants and refugees. MEDLINE (Ovid), Scopus, Web of Science, CABI Global Health and the IGO Custom Search Engine were searched for academic and grey literature published in English, French, or Spanish from inception until September 22nd, 2025. A conceptual framework structured data extraction and thematic analysis of barriers to antibiotic use along the access pathway, including approachability, acceptability, availability, accommodation, affordability, and appropriateness.

**Results:**

This review included 125 studies from an array of geographic locations. Migrants and refugees experience barriers along the continuum of care from both the patient and health-system side, impacting access to, and appropriate use of antibiotics. Limited access to resources, prevalence of certain social norms and values, health literacy and beliefs, and autonomy, can impact healthcare seeking and utilization. Health system barriers, such as location and affordability of services or language barriers, can also limit access and appropriate use of quality-assured antibiotics.

**Conclusion:**

Migrants and refugees face structural, financial, and systemic barriers in accessing and using antibiotics. While globalization processes have shaped the barriers migrants and refugees experience when accessing health services, access pathways are heterogeneous and influenced by the health systems of the host countries, and other contextual, non-health policies and factors. Potential policy solutions to mitigate these barriers include initiatives to address country-of-origin norms and values and improving language accessibility. Improved global policy coordination can also address access challenges for migrants and refugees, as tackling ABR requires collective global action.

**Clinical trial number:**

Not applicable.

**Supplementary Information:**

The online version contains supplementary material available at 10.1186/s12992-026-01188-x.

## Background

Antibiotic resistance (ABR) is a growing global challenge, threatening human health, social well-being, and economic development. In 2021, bacterial resistance emerged as the third leading cause of death, with 1.12 million deaths directly attributable to bacterial resistance worldwide [1]. Globalization has propelled transmission of ABR through expansion in trade, travel, and human and animal migration [2]. The rise in migration has been one of the most significant aspects of globalization as economic, political, and environmental instabilities have led to people moving across borders [3].

Migrant and refugee populations may be particularly vulnerable to the impact of ABR, through increased exposure to drug-resistant pathogens and poor access to healthcare services [4, 5]. The conditions under which migrants and refugees leave their countries of origin, transit to, and live in host countries can lead to increased exposure to infections [6]. Key drivers of infection, such as crowding, poor hygiene, and sanitation may be present in detention centers, refugee camps, and housing for migrant workers, leading to the spread of resistant pathogens [4–7]. Healthcare provision for migrants and refugees can also vary between countries, posing systemic barriers based on legal status; for example, irregular migrants may be ineligible for public health services depending on health systems design in specific country contexts. In addition, informal barriers, such as language and cultural differences, can prevent access to healthcare, and sequentially undermine access to appropriate and effective antibiotics [4, 5]. Such language barriers may reinforce existing cultural differences and instill distrust and promote inappropriate use of antibiotics. Consequently, migrants and refugees are prone to experiencing barriers to accessing timely, affordable, and quality health care services [8–10].

Despite evidence pointing to increased vulnerability to ABR in migrant and refugee populations, much of the existing research examines the risk to exposure and incidence of drug-resistant pathogens and infections in migrants and refugees. The empirical evidence regarding barriers to antibiotic access and appropriate use, two important drivers of resistance in migrant and refugee populations, has not been synthesized yet, highlighting the need for conducting a scoping review to map the research in this area and identify knowledge gaps and critically reflect on global policy implications and actions, both of which have yet to be identified at the global level [11, 12].

## Methods

This rapid scoping review is reported according to the Preferred Reporting Items for Systematic Review and Meta-Analysis extension for Scoping Reviews (PRISMA-ScR) [13]. The reporting guidelines and checklist are included in Appendix Table 1.

### Identification of research questions

The objective of this scoping review is to address the following research question: *What are the barriers faced by migrants and refugees in accessing and appropriately using antibiotics?* The scoping review aims to systematically map the existing evidence, identify evidence gaps, and inform policies and interventions.

### Identification of relevant studies

We employed a three-pronged search strategy to identify evidence through electronic peer-reviewed databases, grey literature, and email requests to regional experts. In consultation with an information specialist at York University, we developed a structured search strategy and systematically searched MEDLINE (Ovid), Scopus and Web of Science for published literature up to 22 September 2025. The search strategy employed for each database is included in Appendix Table 2. The reference lists of included studies were reviewed, and targeted searches were conducted to identify additional literature. A targeted search of the grey literature was completed in two databases, CABI Global Health and The IGO Custom Search Engine, and was performed using key terms such as “refugees”, “migrants”, “antimicrobial resistance”, “antibiotic resistance”, “access”, “misuse” and “barriers”. Database searches were supplemented with email requests for relevant grey literature to experts in World Health Organization (WHO) regional offices and international non-governmental organizations.

### Study selection

Inclusion and exclusion criteria (Table 1) were established in accordance with the review objective. To be included, literature needed to assess migrants’ or refugees’ access or use of antibiotics. This distinction between migrants and refugees reflects the different legal status and associated protection frameworks between refugees and migrants, which can result in differential access to health care. Migrant and refugee populations were defined, and findings were categorized according to the definitions outlined in Table 2; studies that used ethnicity, race, or parents’ place of birth as a proxy for migration status were excluded. We only included studies that investigated antibiotic access or use in relation to humans; however, studies that examined antimicrobial use overall but provided disaggregated antibiotic use data were also included. Studies investigating migrants and refugees’ access and use of antitubercular drugs were not included, as treatment is longer and likely will encounter additional barriers, and other antimicrobials (e.g., antiparasitics, antivirals, antifungals). Articles were included if they were peer-reviewed primary studies of any methodological design, published in English, French, or Spanish, with no date restrictions applied.

**Table 1:** Eligibility criteria for studies

Criteria	Inclusion Criteria	Exclusion Criteria
Population	Human migrants or refugees as defined in Table 2 in any country.	Non-migrant or non-human populations or use unreliable indicators for migrant or refugee statuses^a^.
Context	The publication investigates access or use of antibiotic medication in the context of human consumption.	The publication solely investigates access or use of other types of antimicrobials^b^.
Outcome	The publication assesses access or use of antibiotics^c^, including availability, affordability, consumption, misuse, barriers to use, improving access and use and disruption of access.	The publication reports solely on prevalence of ABR or does not report outcomes of access and use.
Study design	The publication is a primary quantitative or qualitative study, or a review of the literature^d^.	The publication is an editorial or commentary.
Date	Inception of databases to August 2nd, 2023	No date restrictions.
	The publication is written in English, French, or Spanish.	The publication is not written in English, French, or Spanish.

^a^ Studies that used inappropriate proxies for migrant and refugee status (e.g., ethnicity, race, or parents’ place of birth) were excluded

^b^Studies that investigated access or use of antitubercular drugs were also excluded. This is because tuberculosis treatment typically occurs over a longer period and would likely encounter different access or use barriers

^c^ If studies assessed antimicrobials access and use it was included only if findings for antibiotics were reported in disaggregate terms

^d^ The reference lists of systematic reviews and scoping reviews were searched for additional relevant studies but reviews themselves were not included

**Table 2:** Definitions of migrants and refugees

Term	Definition
**Migrant** There is no universally accepted definition of migrant. For the purposes of this study, an international migrant is defined as any person who moved away from their place of usual residence across an international border. The term includes migrants who intend to move permanently or temporarily regardless of their legal status.	
**Labour migrant**	A person voluntarily moving from one country to another for the purpose of employment.
**Irregular migrant**	A person who moves or has moved across an international border outside the laws, regulations, or international agreements governing the sending, transit, and receiving countries and is not authorized to enter or to stay in a country, pursuant to the law of that country and to international agreements to which that country is a party.
**Refugee** Persons who, owing to a well-founded fear of persecution for reasons of race, religion, nationality, conflict, generalized violence, membership of a particular social group or political opinion, are outside the country of their nationality and are unable or, owing to such fear, unwilling to avail themselves of the protection of that country, and consequently requires international protection; or who, not having a nationality and being outside the country of their former habitual residence as a result of such events, are unable or, owing to such fear, unwilling to return to it. This definition refers to individuals who have been formally granted refugee status and legal protection under international or national frameworks, as distinct from asylum seekers who are awaiting such determination.	
**Refugee (camp-based)**	A person residing in a refugee camp or temporary, segregated settlement and facility provided by government and non-governmental organizations, separate from the host population and receive basic healthcare services.
**Refugee (non-camp-based)**	A person currently residing autonomously outside of a temporary facility or refugee camp in urban, peri-urban or rural areas. They are self-sufficient with greater civil and economic freedom and do not receive aid or support from government or non-governmental organizations or rely on public provision for these services.
**Asylum seekers** A person who has fled their own country and is seeking international protection. In countries with individualized procedures, an asylum seeker is someone whose claim has not yet been decided on by the country in which they have submitted it. Living arrangements for asylum seekers can vary widely by country, policies, and resources, for example, can include government-run or non-governmental organization run reception centres, refugee or asylum camps, community-based (non-camp) housing, or shelters and temporary accommodations.	
**Other/ unspecified** Study does not identify the migrant or refugee typology. Migrants that fall outside of the abovementioned typologies. These include persons whose status or means of movement are not clearly or specifically defined in the included studies under international law, such as foreigners admitted for education/training, migrants for settlement (family-, ancestry-, business-based), and retirees.	

All references identified in our database searches and through expert consultations were uploaded and screened in Covidence. Studies were screened in two stages: title and abstract screening and full text screening, with all articles screened by a single reviewer.

To be included as evidence, studies had to clearly identify barriers to accessing or appropriately using antibiotics in migrants or refugee populations. We defined “access” as migrants’ and refugees’ ability to obtain quality-assured antibiotics when necessary, including the physical availability of antibiotics and the affordability of antibiotics [14]. “Appropriate use” includes both patient and prescriber dimensions of appropriate treatment, such as guideline concordance, unnecessary prescriptions, self-medication, and non-adherence to prescribed treatment. We included two types of inappropriate use: (1) unnecessary use, defined as ineffective or unneeded use given the target indication and patient (e.g., on the prescriber-side, ordering use of antimicrobials not needed nor effective for the target indication); and (2) incorrect use, defined as erroneous or non-optimal use of antimicrobials, such as non-adherence to a prescribed therapy by a patient.

### Data charting

Reviewers independently extracted data from the eligible studies using a screening form in Covidence Systematic Review. The following variables were extracted from the included studies: (1) study identifiers: first author, year of publication, country and region of data collection or analysis; (2) study methods: aim of study and study design; (3) study participants: definition of migrant and/or refugee used in study, migrant and/or refugee category; (4) type of antimicrobial and disease context; (5) barriers to access and/or appropriate use outcome.

### Collating, summarising and reporting data

**Fig. 1:**
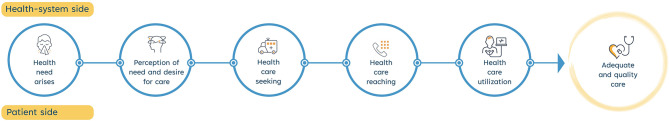
Conceptual framework to antibiotic access and appropriate use along the continuum of care. This figure depicts the stages individuals move through to obtain healthcare, starting with the emergency of a health need to the perception of need, care-seeking, reaching, and utilization, to adequate and quality care from both the patient-side and health-system side

We adapted Levesque et al.’s [14] framework to structure the thematic analysis, which describes access to health care along a continuum (see Fig. 1). A patient’s ability to access care, and thus antibiotics, can be disrupted or facilitated by health system side or patient side factors and barriers at any point along this continuum of care. The continuum is divided into five dimensions that correspond with the accessibility of health services: *(1) Approachability*,* (2) Acceptability*,* (3) Availability and Accommodation*,* (4) Affordability*,* and (5) Appropriateness* [14]. These five dimensions were used to thematically organize the barriers to access and appropriate use of antibiotics, on both the health system and patient sides.

### Stakeholder outreach

We solicited information from the International Committee of the Red Cross, International Organization for Migration, Médecins Sans Frontières, United Nations (UN) Women, UN Refugee Agency, United Nations Children’s Fund (UNICEF), and WHO Regional offices, by email in February and March 2022 for additional documents discussing appropriate antibiotic access and use for migrants and refugees and their barriers.

## Results

**Fig. 2:**
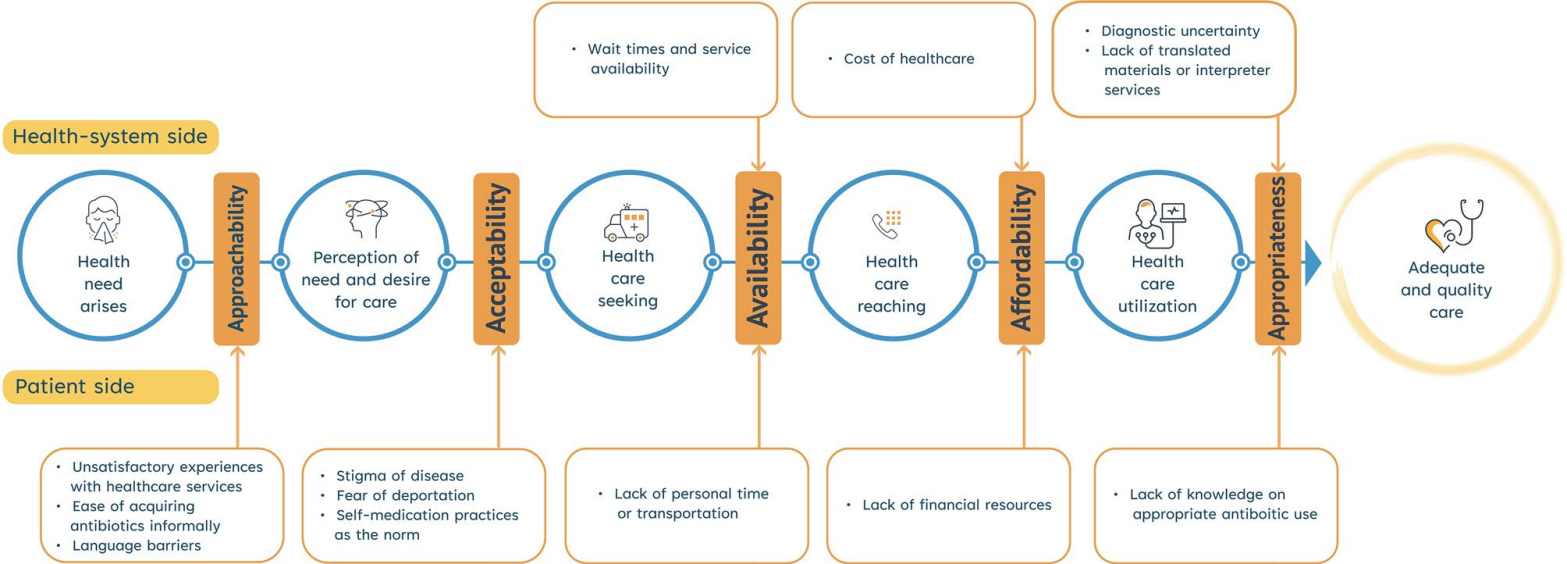
Health-system and patient side barriers to antibiotic access and appropriate use along the continuum of care for migrant and refugee populations. This figure illustrates the barriers along the five key dimensions – approachability, acceptability, availability and accommodation, affordability, and appropriateness – which influence antibiotic access and appropriate use for migrant and refugee populations along the continuum of care from the patient-side and health-system side factors

Of the 6498 records collected, 680 full-text studies met initial title and abstract screening criteria, and 83 met the criteria for full-text inclusion, and an additional 18 snowball searches and 20 additional grey literature and expert sources were included (see PRISMA chart in Appendix Fig. 1), for a total of 121 studies included in the review. The included studies, which met our criteria for full-text inclusion, were from an array of geographic regions, including Europe (*n* = 34), the Americas (*n* = 24), Western Pacific (*n* = 5), Eastern Mediterranean (*n* = 10), Africa (*n* = 3), South-East Asia (*n* = 1), and Global (*n* = 7) (additional general characteristics of the included studies are presented in Appendix Table 3). Across these contexts, barriers on the patient and healthcare side are identified which may disrupt the continuum of care (see Fig. 2), with evidence illustrating that migrants and refugees experienced significant barriers to care linked to migration specific factors, which likely differentially impacted their access to, and appropriate use of antibiotics as compared to host populations.

### Patient side barriers

Access to quality-assured and appropriate antibiotics can be undermined by barriers on the patient side, such as health literacy, health beliefs, social norms and values, autonomy, and access to resources (e.g. transportation and income). Our analysis identified barriers along the patient-centred access to health care pathway (i.e., approachability, acceptability, availability, affordability, and appropriateness) in a total of 48 studies [15–62].

#### Approachability

Approachability refers to people’s capacity to identify services which exist to support their health needs, to reach adequate care, and to impact the health of the individual. For example, the ability for an individual to perceive the need for care is influenced by elements such as health literacy, and knowledge and beliefs towards health [14]. Factors such as complex healthcare systems, previous unsatisfactory experiences with health services, language barriers, and the ability to acquire antibiotics informally, can contribute to making formal healthcare services less approachable for migrants and refugees.

Complexity of the health care system in the host country for migrants and refugees can deter individuals from accessing healthcare [48]. In the United States, migrants, despite having secure jobs, were confused by where to start or who to contact when a healthcare need arose [49]. Similarly, in a study by Nanakali and colleagues [50], lack of familiarity with the United Kingdom’s healthcare system influenced migrants to bring antibiotics from their home country to the United Kingdom.

The evidence suggests that migrants and refugees perceive high-income host countries to be over-regulating antibiotic prescriptions and dispensation as compared with their countries of origin [29, 31, 37, 44, 47]. Migrants and refugees often reported dissatisfying appointments with doctors, especially when antibiotics were not prescribed, believing that practitioners were not taking their illnesses seriously. Consequently, migrants and refugees would refrain from seeking care for new and previously experienced illnesses from their host’s formal health system [16, 23, 25, 26, 28, 29, 51].

Similarly, language barriers deterred migrant and refugee populations from accessing formal healthcare services because of their inability to communicate their health problems [21, 26, 28, 30, 34, 37, 38, 40, 41, 45, 48, 50]. Across geographical contexts, migrants and refugees were also found to have easy access to non-prescribed antibiotics through informal pathways [16, 23, 26, 28, 30, 34, 37, 40, 41, 44–47]. Therefore, unsatisfactory doctor appointments and language barriers drove some migrants and refugees to access antibiotics informally by bringing antibiotics from their origin country, or having them mailed by family or friends [16, 23, 25, 28–30, 32, 33, 44–46]; purchasing them in community stores or pharmacies [17, 23, 25, 28, 30, 33, 41, 44]; using leftover prescriptions [17, 26, 33, 45]; or by ordering them online [45, 47].

#### Acceptability

Acceptability is determined by cultural and social factors which impact an individual’s understanding of whether they should seek healthcare. Pursuing healthcare is also impacted by autonomy and access to resources (e.g. information) to strengthen personal capacity to obtain healthcare [14]. The evidence suggests that migrant and refugee populations’ ability to seek care is shaped by self-medication norms, and stigma of certain infections, discrimination, and fear of deportation for irregular migrants.

Considerable evidence was found which highlighted the common practice of self-medication among refugee and migrant populations [16, 30–32, 52]. Self-medication was especially prevalent among migrants and refugees when antibiotics were available over the counter in their country of origin [21, 25, 38, 41, 44, 46, 49, 52–55]. For example, Latin American migrants in the United States, Pakistani migrants in New Zealand, and Syrian migrants in Turkey, described confidence in self-diagnosing and medicating illnesses without supervision, for themselves and their children, based on past experiences [16, 31, 53].However, evidence highlighted inappropriate use of antibiotics through self-medication practices, such as antibiotics being used to treat viral infections [23, 26, 31, 49, 52, 54], non-infectious diseases [37, 43], or through inadequate dosages [37, 43, 52]. While the majority of studies did report self-medication practices among migrants and refugees, in Hong Kong, migrants were less likely to self-medicate with antibiotics in comparison to native individuals [55].

Stigma, discrimination, and fear of deportation was also found to increase rates of self-medication practices among refugees and migrants. For instance, cultural and social views on sexually transmitted infections and tuberculosis were found to deter migrants from seeking healthcare through formal pathways, delay care, or influence individuals to opt for informal care and self-medication [15, 38, 44, 54]. Refugees in Colombia felt distrust towards healthcare providers as they experienced hostile behaviours from workers and believed that they received lower standards of care due to their migrant status [54]. Additionally, irregular migrant status or lack of legal registration interfered with refugees’ and asylum seekers’ ability to seek formal health care [21, 38, 41, 47, 54, 56, 57].

#### Availability and accommodation

Availability and accommodation indicate that health services can be reached physically and punctually. This depends on an individual’s mobility, where they can access transportation, have occupational flexibility, and the knowledge to allow them to reach healthcare facilities [14]. Availability and accommodation of health services for migrants and refugee populations was predominantly impeded by insufficient access to healthcare services, a lack of personal time and limited transportation means [25, 27, 37, 41, 47, 50, 53, 57, 58]. Indeed, one Australian study found that the dominant barrier by migrants to consulting a medical practitioner was time constraints [27].

#### Affordability

Personal capacity to spend financial resources on appropriate healthcare services refers to the affordability of care, where an individual’s capacity is related to the expenses associated with receiving health services (e.g., care and transportation costs, and loss of income) [14]. For migrants and refugees, affordability was shown to deter formal healthcare access [49, 52, 54]. A study on refugees living in Colombia, Yemen, and Uganda, all reported that price influences their decision to access healthcare and purchase appropriate antibiotics [54]. Similarly, migrants from Mexico living in the United States detailed their preference to receive treatment administered by a doctor; however, a lack of health insurance and limited emergency funds resulted in self-medication practices [25]. Limited financial resources also influenced whether migrants in the United Kingdom would follow a physician’s antibiotic regimens, for example, if they did not have the funds to purchase antibiotics prescribed. This often led to purchasing antibiotics in their home countries and bringing them back to the United Kingdom to self-medicate when ill [50].

#### Appropriateness

Appropriateness signifies the suitability of services for a person’s needs, as well as adequate and timely treatment. Concurrently, appropriate healthcare is dependent on one’s opportunity to utilize such services [14]. Limited knowledge on ABR – which is not exclusive to migrant and refugee populations – can influence lack of adherence to antibiotic treatments [20, 36] or use of antibiotics without prescriptions [17–19, 21–25, 28, 38, 39, 41, 44, 45, 52, 59–62]. While having a regular doctor was found to improve treatment adherence among Asian migrants in the United States [20], migrants and refugees living in host countries across differing regions frequently reported feeling better as a central reason to discontinuing treatment early [26, 34, 42]. Other reasons to prematurely discontinue treatment among refugees and migrants included: feeling uneasy about using antibiotics for long periods; believing antibiotics are too strong for one‘s body [46]; and preferring injectable antibiotics because they were perceived to be more effective [23, 25, 37, 38, 41, 44].

### Health system side barriers

Access to quality and appropriate antibiotics is also dependent on the supply side of health systems, with factors such as location and cost impacting patient care. The evidence review produced a total of 51 studies [16, 17, 20, 21, 23, 25, 27, 30–32, 34, 36–38, 41, 63–98] which identified barriers within the health system that disproportionally impact migrant and refugee populations. The principal entry points along Levesque et al.’s [14] framework that produced obstacles include the availability, affordability, and appropriateness dimensions, with key themes presented below.

#### Availability

On the health systems side, availability refers to the physical existence of health facilities, encompassing the factors such as the density, concentration, distribution, accessibility by transportation, duration and flexibility of opening hours, and presence and qualification of healthcare providers [14]. Four interrelated principal characteristics in the published literature presented potential barriers from the health system side: density of facilities and presence of ample healthcare providers; and waiting times and service availability.

For refugees living in camps, and asylum seekers that have no legal entitlement to care in host countries, specialized national programs may exist to provide healthcare services. These national programs often have limited resources and capacities, impacting the availability of care in some settings [52, 53, 63, 64, 77, 91, 92]. This is further compounded by large influxes of migrants and refugees utilizing these specialized services as overcrowding or outbreaks reduces the number of space and practitioners available to provide care [36]. Similarly, wait times can also be extensive in health centres run by humanitarian organizations, and were found to explain self-medication practices for some refugees [17]. When accessing formal healthcare is challenging in these settings, informal markets for healthcare can also emerge. For example, in Shatila, a refugee center in Lebanon, refugees used these informal pharmacies to purchase cheap antibiotics quickly without a prescription [62].

Despite variations in entitlement of healthcare, healthcare professionals commonly reported challenges with providing care for irregular migrants living in Europe because of the patient’s limited funds, or the administrative requirements, and practices and procedures of the healthcare facility. In response to these challenges, physicians would refer irregular migrants to other centres [64].

Long wait times and service unavailability in health facilities also created availability barriers for migrant and refugee populations with legal entitlement to healthcare within their host country [16, 17, 20]. For example, Pakistani migrant mothers in New Zealand reported that they experienced challenges in accessing healthcare because of the unavailability of appointments and long wait times, which can push them to self-medicate [16].

#### Affordability

Affordability from the health systems side encompasses the direct and indirect costs of health services, and coverage by insurance schemes [14]. Similar to legal entitlement to care, coverage of healthcare costs by insurance is often associated with legal status, and migrants or asylum seekers without formal recognition may thus be excluded from such benefits [72, 88]. Moreover, legal status permitting the usage of national health benefits does not always translate into migrants and refugees being aware and claiming these public benefits. The published literature documented the low affordability and inability to access health insurance coverage as a principal factor impeding healthcare and prescription service utilization by migrants and refugees in their host countries [16, 21, 23, 25, 26, 30–32, 37, 38, 41, 56, 62, 71, 77, 78, 84, 85, 93]. Specifically, in the United States, affordability proved to be a significant barrier for migrant and refugee populations as universal health programs are difficult to access for these groups, leading many to adopt self-medication practices, or travel to their home countries for treatment [21, 23, 25, 30–32, 37, 38, 41, 49, 77]. In the United States, even migrants with healthcare insurance still brought antibiotics from their home country to self-medicate when needed, to avoid the high and unpredictable cost of visiting an emergency department for health care [49]. However, studies on healthcare services provided by non-governmental organizations in camp-like settings were found to experience fewer barriers to accessing care because of the lower costs associated with treatment, compared to refugees not living in the camps in the same region [65, 86, 87].

#### Appropriateness

Appropriateness accounts for the health systems’ ability to provide correct and timely services for the needs of the patients [14]. The published literature has highlighted three health system barriers to appropriate care: diagnostic uncertainty; lack of translated services; and substandard medications as producing limited access to quality antibiotics and unnecessary and incorrect use of antibiotics for migrant and refugee populations.

In comparison to host populations, evidence suggests that migrants, refugees, and asylum seekers are either more likely [51, 59, 67, 70, 74, 79, 81, 94, 95], as likely [73, 96], or less likely [97] to be prescribed antibiotics unnecessarily or incorrectly for infections [68, 75]. There is limited evidence explaining barriers to appropriate antibiotic prescriptions; however, a study by Moro et al. [81] found pediatricians reported diagnostic uncertainty – inappropriately prescribing antibiotics because of the inability to diagnose a patient’s health problem – as an explanation for unnecessary prescriptions. Pediatricians were found to be more likely to prescribe children with at least one foreign-born parent antibiotics for infections that were unlikely to be bacterial [81]. Similar findings were reported in a German study where migrants, or patients with migrant parents, had an increased likelihood of being prescribed antibiotics for colds or upper respiratory tract infections [70]. In refugee centers, health care providers have also been documented to overprescribe antibiotics [57, 58, 62]. In a rural refugee settlement in Uganda, antibiotics prescribed to refugees did not follow the national treatment guidelines and were often over prescribed to patients [57]. Overprescribing by physicians may be to compensate for the language barriers, or for the risk for infections from living conditions in the refugee camps [58, 98].

Language barriers may contribute to diagnostic uncertainty via miscommunications of symptoms between healthcare providers and patients, leading to unclear diagnoses [56, 76, 85]. Unclear antibiotic instructions resulting from language barriers can also impact appropriate healthcare [56]. For example, a study on Turkish migrants living in Europe found that doctors had to consult staff, other customers, or relatives to help translate dosages and medication side-effects; and antibiotic instructions were also not typically available in Turkish, interfering with appropriate healthcare for foreign-born populations [34].

Substandard and falsified antibiotics also present an obstacle to appropriate healthcare for migrants and refugees. Stock-outs of antibiotics are common in emergency settings (e.g., in refugee camps), especially following an influx of arrivals, overcrowding, or outbreaks, making substandard or falsified medicines more accessible when quality-assured medicines are unavailable or unaffordable [36, 66–69, 80, 82, 83]. A study on refugee settlements in Uganda found that approximately 32% of antibiotics, including essential antibiotics, were stocked out on a given day. When standard procurement of antibiotics is unfeasible, healthcare facilities in humanitarian settings may acquire medicines from less reliable, illegitimate, or alternative suppliers; or prescribe other antibiotics not appropriate for the infection [68, 89, 90].

## Discussion

Globally, more than five million deaths could be prevented annually by providing comprehensive access to antibiotics [99]. Since the 2000s, there has been an increase in antibiotic consumption globally. Despite recording progress in facilitating access to antibiotics over the past decades through pharmaceutical supply chains’ expansion of the distribution of antibiotics, access to appropriate and effective antibiotics remains deeply inequitable due to existing socio-economic, legal, and structural barriers. Amidst growing socio-economic inequalities globally which further heighten access challenges within health systems, this is especially prevalent for migrant and refugee populations. Barriers to populations, especially migrants and refugees, to accessing and appropriately using antibiotics, operate at both the patient and health system levels and are further intensified by lack of transnational healthcare coordination and poorly executed migration governance.

On the patient side, our findings highlight how previous health care related experiences — often shaped by globalization-related displacement, conflict, and exclusion from formal health systems — impact health seeking behaviors through normative predispositions and culturally shaped attitudes. This may lead migrants and refugee populations to rely on informal access pathways undermining appropriate use and limit what health care interventions are considered acceptable. Additionally, restrictive immigration and asylum policies may discourage irregular migrants from seeking formal medical care, further exacerbating health inequities. On the health systems side, our findings underscore the role of access barriers, with the literature documenting low affordability and inability to access health insurance coverage, which vary in different country contexts, as principal factors impeding healthcare and prescription service utilization by migrants and refugees. Furthermore, diagnostic uncertainty, lack of translated services, and substandard medications reinforce limited access to quality antibiotics and may lead to unnecessary and incorrect use of antibiotics for migrant and refugee populations. However, while we have been able to identify important barriers to antibiotic access and use, the evidence base for the assessment of refugees’ and migrants’ access to antibiotics remains severely limited, with a concerning paucity of studies covering low- and middle-income countries or refugee camp settings as part of our data sample. The available data mainly focuses on high-income contexts, where differences in health systems, legal entitlements, and drug reimbursement programs contribute to variable access levels among refugees and migrant populations. This research gap underscores the need for targeted funding and concerted efforts to address these knowledge deficiencies in the Global Research Agenda for Antimicrobial Resistance (AMR) – resistance to infections not only caused by bacteria, but also parasites, viruses, and fungi – to protect human health [100].

Our findings have important policy implications and recommendations at the global and national level (see Table 3). At the global level, structural policy changes are needed as mitigating ABR requires global cooperation and cannot be addressed by individual countries alone. To better understand the health needs of refugee and migrant populations and their heightened vulnerabilities to ABR, including broader susceptibility to antimicrobial resistance (AMR), and their unique barriers to health care access, global AMR surveillance systems need to be implemented. Global data collection can also identify and support research into knowledge gaps to better address barriers to health care for refugee and migrant populations. Our findings also demonstrate the importance of building global governance for AMR actions whereby the needs of refugees and migrants are aligned and integrated into WHO global action plans to promote an equity-focused framework for AMR action. Lastly, global governance for AMR needs to more broadly promote equitable antibiotic access and appropriate use in low-resource countries. This should include the establishment of globally redistributive financing mechanisms to ensure antibiotic access for populations with heightened ABR vulnerabilities. One such example is the development of a Global AMR Conservation Fund [101].

At the national level, three key implications have been identified. First, the role of country-of-origin-based norms and values in decision-making surrounding inappropriate antibiotic use highlights the importance of education and improving migrants’ and refugees’ knowledge of antibiotics through community-based initiatives. Similarly, it is critical to incorporate migrant- and refugee-sensitive cultural competency training for healthcare workers to strengthen care for populations that are more vulnerable to accessing and appropriately using antibiotics. Second, access barriers related to language mean that some policy interventions should focus on facilitating access to care by improving language accessibility (including through interpreters or bilingual staff) and health literacy for refugees and migrants. Third, migrant status intersects with and exacerbates the typical determinants of access to care, such as socioeconomic position [102]. Even where care may be affordably priced, patient ability to pay is not guaranteed, with limited economic opportunities for migrants and refugees. For irregular migrants, lack of coverage may be coupled with exploitative practices at work, which reduce both their earning potential and access to work-provided health insurance [103]. This makes removing systemic barriers to care based on legal status, as well as ensuring that essential, quality-assured antibiotics are affordable through implementation of equity-focused pre-payment programs a crucial aspect of an effective policy response [104, 105].

**Table 3:** Policy recommendations to improve antibiotic access and use among migrants and refugees

Global policy recommendations	
Establish a global AMR surveillance system	Implement a global surveillance system which systematically includes migrants and refugees to fill knowledge gaps and support the development of targeted interventions to address health barriers in these populations.
Strengthen global AMR governance	Include refugees and migrants into WHO’s Global Action Plan on AMR to promote an equity-focused framework for coordinated action.
Advance equitable antibiotic access and stewardship	Strengthen global governance mechanisms to promote appropriate antibiotic use and availability in low-resource settings by implementing redistributive financing models, building on and scaling up WHO’s Expanding Sustainable Access to Antibiotics (SECURE) initiative [105].
**National policy recommendations**	
Develop migrant-and refugee-sensitive cultural competency training	Integrate cultural competency training into pre-service education and continuing professional development for healthcare workers to strengthen their skills to understand diverse health beliefs, communications norms, and healthcare seeking behaviours among migrants and refugees.
Improve communication pathways between healthcare providers and migrant and refugee populations	Expand language support, culturally tailored health education, and health literacy programs to promote access to and appropriate antibiotic use.
Remove systemic barriers to health care linked to legal status	Adopt equity-focused financing mechanisms to make essential, quality-assured antibiotics accessible to all individuals regardless of legal status.

This scoping review has several limitations. To address the scarcity of existing academic literature on migrant and refugee access and use of antibiotics, we used triangulation of data sources and included various grey literature sources in the dataset. This scoping review was conducted as a rapid review, and due to time constraints, abstract and full-text screening was performed by a single reviewer. This may have introduced selection bias. To reduce the risk of bias while using a single-reviewer approach, we developed a study protocol, clearly defined inclusion and exclusion criteria, and used cross-checking of coded information for a small subset of data extraction results by a second reviewer. Additionally, due to limitations in the language fluency of the research team, we only included studies published in English, French, and Spanish. Lastly, the included articles were not appraised critically due to time constraints and the lack of available tools to appropriately appraise non-intervention studies. We suggest future scoping reviews apply quality assessment tools to strengthen review methodology. Additional studies should also assess migrants and refugees separately, and compare and contrast their main challenges over time to identify more specific health needs and trends for each population group, as well as study in more depth the context-specific factors that determine access to and appropriate use of antibiotics.

## Conclusion

Our scoping review found that refugees and migrants encounter significant barriers in obtaining and using antibiotics effectively throughout their healthcare journey. Globalization processes have shaped these challenges by influencing pharmaceutical supply chains, migration policies, and health system fragmentation, often exacerbating inequities. Due to exclusion from formal health services, displaced populations frequently rely on informal antibiotic access pathways, increasing the risk of inappropriate antibiotic use and ABR emergence. Previous experiences, cultural norms, and personal preferences may create a negative perception towards formal healthcare, while legal and financial obstacles further undermine adequate access to antibiotics. Given the heightened susceptibility of refugees and migrants to ABR while facing unique socio-economic challenges, it is imperative to enhance global efforts to facilitate safe and sustainable access to antibiotics for migrants and refugees. This means aligning AMR action plans with those addressing migrant and refugee health and broader healthcare accessibility, and better coordinating refugee and asylum policies across borders to engender improved healthcare access. To promote equity and accessibility in the use and distribution of antibiotics, solutions must focus on better global coordination to facilitate the implementation of policies that remove socio-economic barriers to access, as well as development of targeted programs that specifically address the needs of populations with heightened ABR vulnerabilities, such as refugees and migrants.

## Supplementary Information

Below is the link to the electronic supplementary material.

Supplementary Material 1: Appendix Table 1. Preferred reporting items for systematic reviews and meta-analyses extension for scoping reviews (PRISMA-ScR) checklist.

Supplementary Material 2: Appendix Table 2. Search strategies.

Supplementary Material 3: Appendix Table 3. Summary of included studies.

Supplementary Material 4: Appendix Fig. 1. Prisma chart.

## Data Availability

All data generated or analysed during this study are included in this published article and its supplementary information files.

## Abbreviations

ABR: Antibiotic resistance
AMR: Antimicrobial resistance
WHO: World Health Organization
