# Supplementary material for: A rapid scoping review of antibiotic access and use barriers among refugee and migrant populations
Source: Global Health. 2026 Jan 29;22:20. doi: 10.1186/s12992-026-01188-x (PMC12874966; doi:10.1186/s12992-026-01188-x)
Supplement: Supplementary file 2 — Supplementary Material 2: Appendix Table 2. Search strategies. [file 12992_2026_1188_MOESM2_ESM.docx]

**APPENDIX TABLE 2. SEARCH STRATEGIES**

| **DATABASE** | **TERMS** |
| --- | --- |
| **OVID Medline** | **Core Search Terms** |
|  | 1. (migrant? Or refugee? Or emigra* or immigra* OR migrat* or asylum or alien? Or foreigner? Or foreign-born or foreignborn Or (Displaced ADJ0 (individual? OR person? OR people? OR population?)) or (countr* ADJ2 (birth OR origin))).mp 2. "Emigrants and Immigrants"/ OR "Transients and Migrants"/ OR "Emigration and Immigration"/ OR Refugees/ OR "Human Migration"/ 3. (AMR OR XDR OR XDR-TB OR XDRTB OR MDR OR MDR-TB OR MDRTB OR DRI OR (resistan* ADJ2 (drug? OR multi-drug OR multidrug OR extensively-drug OR infection?)) OR anti-viral? OR antiviral? OR anti-fungal? OR antifungal? OR anti-bacterial? OR antibacterial? OR anti-biotic? OR antibiotic? OR anti-infective? OR antiinfective? OR anti-microbial? OR antimicrobial? OR anti-parasitic? OR antiparasitic? OR non-susceptib* or nonsusceptib* OR ESBL OR extended spectrum beta-lactamase OR MRSA or methicillin-resistant staphylococcus aureus).mp 4. Drug Resistance, Microbial/ OR Drug Resistance, Viral/ OR Drug Resistance, Fungal/ OR Drug Resistance, Multiple/ OR Drug Resistance, Bacterial/ OR Drug Resistance, Multiple, Bacterial/ OR Anti-Infective Agents/ OR Anti-Bacterial Agents/ OR Antifungal Agents/ OR Antiparasitic Agents/ OR Antiviral Agents/ OR Antitubercular Agents/ OR Methicillin-Resistant Staphylococcus aureus/ 5. (access* OR afford* OR availab* OR sale? OR market? OR supply OR supplies OR funding OR pric* OR cost* OR reimbursement).mp 6. Health Services Accessibility/ OR Delivery of Health Care/ OR Patient Care/ OR Healthcare Disparities/ OR Needs Assessment/ OR Culturally Competent Care/ OR “Health Services Needs and Demand”/ 7. (stewardship OR ((“use” OR usage) ADJ1 (irrational OR rational OR nonrational OR no#-rational OR inappropriate OR appropriate OR no#-appropriate OR off-label* OR off-li?en?e? OR unapprove? OR non-indicate? OR nonindicate?)) OR ((Substandard* OR counterfeit* OR fals*) ADJ1 (drug? OR medication?)) OR overuse OR misuse OR consum* OR prescri* OR overprescri*)~~.~~mp |
|  | **Search Limits** |
|  | 1. Health Services Misuse/ or Medical Overuse/ or Health Knowledge, Attitudes, Practice/ or Potentially Inappropriate Medication List/ or Inappropriate Prescribing/ or Medication Errors/ or Diagnostic Errors/ or "Off-Label Use"/ or Substandard Drugs/ or Counterfeit Drugs/ or "Treatment Adherence and Compliance"/ or (Practice Patterns, Physicians'/) 2. Exp animals/ not humans/ 3. Drug-resistance, neoplasms/ or Anti-neoplastic agents/ or neoplasms/ or brain neoplasms/ or colonic neoplasms/ or Cell proliferation/ or cell movement/ or Angiogenesis inhibitors/ 4. ((1 OR 2) AND (3 OR 4) AND (5 OR 6 OR 7 OR 8)) not (9 or 10) |
| **SCOPUS** | **Core Search Terms** |
|  | 1. (TITLE-ABS-KEY((migrant OR refugee OR emigra* OR immigra* OR migrat* OR asylum OR alien OR foreigner OR "foreign-born" OR foreignborn OR (displaced W/0 (individual OR person OR people OR population)) OR (countr* W/2 (birth OR origin)))) 2. TITLE-ABS-KEY((amr OR xdr OR “xdr-tb” OR xdrtb OR mdr OR “mdr-tb” OR mdrtb OR dri OR (resistan* W/1 (drug OR “multi-drug” OR multidrug OR “extensively-drug” OR infection)) OR anti-viral OR antiviral OR anti-fungal OR antifungal OR anti-bacterial OR antibacterial OR anti-biotic OR antibiotic OR anti-infective OR antiinfective OR “anti-microbial” OR antimicrobial OR “anti-parasitic” OR antiparasitic OR “non-susceptib*” OR nonsusceptib* OR esbl OR “extended spectrum beta-lactamase” OR mrsa OR “methicillin-resistant staphylococcus aureus”)) 3. TITLE-ABS-KEY(((access* OR afford* OR availab* OR sale OR market OR supply OR supplies OR funding OR pric* OR cost* OR reimbursement) OR (stewardship OR (("use" OR usage) W/1 (irrational OR rational OR nonrational OR “no?-rational” OR inappropriate OR appropriate OR “no?-appropriate” OR off-label* OR off-li?en?e? OR unapprove? OR “non-indicate?” OR nonindicate?)) OR ((Substandard* OR counterfeit* OR fals*) W/1 (drug OR medication)) OR overuse OR misuse OR consum* OR prescri* OR overprescri*)))) |
|  | **Search Limits** |
|  | 1. ( LIMIT-TO ( EXACTKEYWORD,"Antibiotic Agent" ) OR LIMIT-TO ( EXACTKEYWORD,"Migration" ) OR LIMIT-TO ( EXACTKEYWORD,"Immigrant" ) OR LIMIT-TO ( EXACTKEYWORD,"Tuberculostatic Agent" ) OR LIMIT-TO ( EXACTKEYWORD,"Antibiotic Resistance" ) OR LIMIT-TO ( EXACTKEYWORD,"Antibiotic Therapy" ) OR LIMIT-TO ( EXACTKEYWORD,"Antitubercular Agents" ) OR LIMIT-TO ( EXACTKEYWORD,"Anti-Bacterial Agents" ) OR LIMIT-TO ( EXACTKEYWORD,"Migrant" ) OR LIMIT-TO ( EXACTKEYWORD,"Multidrug Resistance" ) OR LIMIT-TO ( EXACTKEYWORD,"Emigrants And Immigrants" ) OR LIMIT-TO ( EXACTKEYWORD,"Multidrug Resistant Tuberculosis" ) OR LIMIT-TO ( EXACTKEYWORD,"Drug Resistance" ) OR LIMIT-TO ( EXACTKEYWORD,"Emigration And Immigration" ) OR LIMIT-TO ( EXACTKEYWORD,"Refugee" ) OR LIMIT-TO ( EXACTKEYWORD,"Health Care Access" ) OR LIMIT-TO ( EXACTKEYWORD,"Tuberculosis, Multidrug-Resistant" ) OR LIMIT-TO ( EXACTKEYWORD,"Immigration" ) OR LIMIT-TO ( EXACTKEYWORD,"Antivirus Agent" ) OR LIMIT-TO ( EXACTKEYWORD,"Transients And Migrants" ) OR LIMIT-TO ( EXACTKEYWORD,"Antiviral Therapy" ) OR LIMIT-TO ( EXACTKEYWORD,"Health Care Policy" ) OR LIMIT-TO ( EXACTKEYWORD,"Health Services Accessibility" ) OR LIMIT-TO ( EXACTKEYWORD,"Refugees" ) OR LIMIT-TO ( EXACTKEYWORD,"Antiviral Agents" ) OR LIMIT-TO ( EXACTKEYWORD,"Antibiotics" ) OR LIMIT-TO ( EXACTKEYWORD,"Antibiotic Prophylaxis" ) OR LIMIT-TO ( EXACTKEYWORD,"Health Program" ) OR LIMIT-TO ( EXACTKEYWORD,"Health Service" ) OR LIMIT-TO ( EXACTKEYWORD,"Antibiotic Sensitivity" ) OR EXCLUDE ( EXACTKEYWORD,"Nonhuman" ) OR EXCLUDE ( EXACTKEYWORD,"Animals" ) OR EXCLUDE ( EXACTKEYWORD,"Cell Migration" ) OR EXCLUDE ( EXACTKEYWORD,"Animal" ) OR EXCLUDE ( EXACTKEYWORD,"Cell Proliferation" ) ) 2. 1 AND 2 AND 3 AND 4 |
| **WEB OF SCIENCE** | **Core Search Terms** |
|  | 1. (migrant$ Or refugee$ Or emigra* or immigra* OR migrat* or asylum or alien$ Or foreigner$ Or foreign-born or foreignborn Or (Displaced NEAR/0 (individual$ OR person$ OR people$ OR population$)) or (countr* NEAR/2 (birth OR origin))) (TOPIC) 2. (AMR OR XDR OR XDR-TB OR XDRTB OR MDR OR MDR-TB OR MDRTB OR DRI OR (resistan* NEAR/2 (drug$ OR multi-drug OR multidrug OR extensively-drug OR infection$)) OR anti-viral$ OR antiviral$ OR anti-fungal$ OR antifungal$ OR anti-bacterial$ OR antibacterial$ OR anti-biotic$ OR antibiotic$ OR anti-infective$ OR antiinfective$ OR anti-microbial$ OR antimicrobial$ OR anti-parasitic$ OR antiparasitic$ OR non-susceptib* or nonsusceptib* OR ESBL OR “extended spectrum beta-lactamase” OR MRSA or “methicillin-resistant staphylococcus aureus”) (TOPIC) 3. (access* OR afford* OR availab* OR sale$ OR market$ OR supply OR supplies OR funding OR pric* OR cost* OR reimbursement) OR (stewardship OR ((“use” OR usage) NEAR/1 (irrational OR rational OR nonrational OR no?-rational OR inappropriate OR appropriate OR no?-appropriate OR off-label* OR off-li?en?e$ OR unapprove$ OR non-indicate$ OR nonindicate$)) OR ((Substandard* OR counterfeit* OR fals*) ADJ1 (drug$ OR medication$)) OR overuse OR misuse OR consum* OR prescri* OR overprescri*) (TOPIC) |
|  | **Search Limits** |
|  | 1. NOT Web of Science Categories: Oncology or Biochemistry Molecular Biology or Cell Biology or Veterinary Sciences or Zoology or Plant Sciences or Marine Freshwater Biology 2. 1 AND 2 AND 3 AND 4 |
