# Supplementary material for: A rapid scoping review of antibiotic access and use barriers among refugee and migrant populations
Source: Global Health. 2026 Jan 29;22:20. doi: 10.1186/s12992-026-01188-x (PMC12874966; doi:10.1186/s12992-026-01188-x)
Supplement: Supplementary file 3 — Supplementary Material 3: Appendix Table 3. Summary of included studies. [file 12992_2026_1188_MOESM3_ESM.docx]

**APPENDIX TABLE 3. SUMMARY OF INCLUDED STUDIES**

|  | **Author, Year, and Country** | **Study Aims** | **Study Design** | **Migrant Category** | **Gender Sub-Analysis** | **Barrier Pathway** | **Outcome of Interest** | **Key Findings** |
| --- | --- | --- | --- | --- | --- | --- | --- | --- |
|  | Akhtar, 2021, New Zealand | To explore self-medication practices of Pakistani mothers for their children. | Qualitative research | Other/unspecified | Yes | Approachability, Acceptability, Availability, Affordability | Knowledge and experiences regarding self-medication behaviours | - Pakistani mothers use informal methods to source antibiotics, *e.g.,* bringing medicine from Pakistan before moving, or from friends and relatives who visit. - Mothers resort to self-medication when they do not receive a prescription from their practitioner or because of financial barriers. |
|  | Al Baz, 2018, Jordan | To study antibiotic knowledge, attitude and behaviour of Palestine refugees attending UNRWA health centers in Jordan. | Cross sectional study | Refugees (camp/non-camp based) | Yes | Approachability, Appropriateness, Availability | Knowledge, attitudes, and behaviours regarding antibiotic use | - Refugees experience access barriers to UNRWA health centers, *e.g.* long waiting times and high costs, which drives self-medication practices. - Survey results reveal antibiotic misuse through sharing or over-the-counter (OTC) purchases. |
|  | Aljadeeah, 2024, Europe | To assess asylum seekers, refugees, and undocumented migrants access to essential medicines. | Qualtiative research | Refugees (camp/non-camp based); asylum seekers; irregular migrants | No | Appropriateness | Access to antibiotics at the stages of departure, transit, and deportation. | - Asylum seekers in Germany have higher rates of broad-spectrum antibiotic use compared to host population. - Irregular migrants take higher doses or use them outside prescribed schedules, however this is not exclusive to migrants, but specific factors such as limited affordability, availability, and legal barriers to healthcare can drive inappropriate antibiotic use. |
|  | Al Kady, 2024, Lebanon | To study whether adding a urine culture diagnostic test to urinary tract infection diagnosis limited unnecessary antibiotic prescriptions in pregnant women from refugee camps in Lebanon. | Cross sectional study | Refugees (camp based) | Yes | Appropriateness | Prescription rates for refugees that are pregnant | - A high proportion of refugees that are pregnant are inappropriately prescribed antibiotics. |
|  | Al Karmi, 2024, Jordan | To assess the association between menstrual care and urinary tract infections among refugee women. | Cross sectional study | Refugees (camp based) | Yes | Appropriateness | Causes and treatments for refugee women with urinary tract infections. | - Women in the refugee camp took anitbiotics without prescription for urinary tract infections. |
|  | Alkirawan, 2022, Netherlands | To explores the perspectives and expectations of Syrian refugees in the Netherlands on antibiotic use and prescribing the Dutch primary care system. | Qualitative research | Refugees (unspecified) | Yes | Approachability, Availability, Appropriateness | Refugee perspectives towards antibiotic use/prescribing | - Some refugees may become more cautious with antibiotic use in host countries with stricter regulations. - Some refugees may experience dissatisfaction with the difficulty in accessing antibiotics through the Dutch healthcare system. - Cultural barriers, lack of awareness of AMR, and beliefs about immunity also hinder appropriate antibiotic use. |
|  | Banatvala, 1994, United Kingdom | To compare metronidazole resistance in H. pylori isolates from United Kingdom's Indigenous population to those born in other countries. | Cohort study | Other/unspecified | Yes | Appropriateness | Prescription rates | - Foreign born patients and Bangladeshi patients were more likely to receive an antibiotic prescription than United Kingdom patients. |
|  | Bonniface, 2021, Uganda | To evaluate antibiotic prescribing practices in a rural district in Uganda. | Cross sectional study | Refugees (camp based) | No | Appropriateness | Number/percentage of diagnoses or prescriptions in accordance with guidelines | - Many diagnoses and prescriptions failed to comply with the national clinical guidelines. - Antibiotic prescriptions were provided when they were not indicated. |
|  | Bunuel Alvarez, 2004, Spain | To determine parents' level of knowledge and beliefs on appropriate antibiotic use in children (<6 years). | Cross sectional study | Other/unspecified | No | Appropriateness | Adequate level of knowledge on the proper use of antibiotics (%) in relation to other independent variables (gender, education level, immigrant status, etc.) | - The immigrant population was found to have a lower level of adequate knowledge on the use of antibiotics (9.4%) compared with the native-born population (33.8%). |
|  | Carrasco-Garrido, 2009, Spain | To determine the prevalence of the consumption of medications, prescribed and self-medicated, among the immigrant population in Spain. | Cross sectional study | Labour migrants; Other/unspecified | Yes | Appropriateness, Approachability | Prevalence of medication consumption | - There was not a statistically significant difference in the consumption of antibiotics in the host population compared to immigrants. |
|  | Cho, 2006, United States | To study the rate at which H. pylori infection is treated in an immigrant cohort after diagnosis by esophagogastro-duodenoscopy (EGD). | Cohort study | Other/unspecified | No | Appropriateness, Availability | Proportion of patients that received a prescription at follow up and the proportion of patients with a prescription that completed treatment | - Despite providing EGD results and clear recommendations for treatment, 10 of 49 patients did not receive a prescription at follow-up. - 44% of the patients that received a prescription failed to complete their treatment. The significant barrier to treatment was having a "real doctor." |
|  | Coffman, 2008, United States | To explore how Latino immigrants obtain and use prescription medications without accessing the formal healthcare system. | Qualitative research | Labour migrants; Irregular migrants | No | Approachability, Acceptability, Appropriateness, Affordability | Focus group discussion with Latino immigrants to explore how they obtain and use prescription medications without accessing formal healthcare | - Study participants were found to self-medicate based on their experience of symptoms, past experiences and advice from family and friends. - Barriers formal healthcare for antibiotics included: costs, communication issues, and fear of deportation. |
|  | Comelli, 2024, Global | To assess the determinants that increase refugees, asylum seekers, and internally displaced persons risk of antimicrobial resistance. | Qualitative research | Refugees (camp/noncamp based); asylum seekers | No | Acceptability, Availability, Appropriateness | Narrative review of the determinants of AMR in country of origin, transit centres, and in host country among refugees, asylum seekers, and internally displaced persons. | - Migrants and asylum seekers often experience poor living conditions with overcrowding, poor sanitation, and limited healthcare access. - Fear of deportation for irregular migrants deter them from seeking healthcare, often leading to self-medication. - Helalthcare facilities in refugee camps in Uganda inappropriately prescribed antibiotics. |
|  | Cotugno 2025, Global | To examine the relationship between migration and AMR and healthcare access challenges. | Qualitative research | Labour migrants; refugees (camp/noncamp based); asylum seeker | Yes | Acceptability, Availability, Appropriateness | Narrative review of factors leading to AMR amongst migrant populations. | - Living conditions in refugee camps and migrant shelters, in combination with challenges to accessing health care, can lead to self-medication practices. |
|  | D'Aloja, 2022, Italy | To describe the prescription pattern of medicines among pregnant immigrant women from high migratory pressures compared to Italian women and identify potential inappropriate prescription areas among the two populations. | Cross sectional study | Other/unspecified | Yes | Appropriateness | Distribution of total prescriptions reimbursed by the NHS preterm, during pregnancy, and post-term. | - Migrant women are less likely to receive prescriptions, including for antimicrobials, than Italian women throughout pregnancy. |
|  | Děger, 2023, Turkey | To assess factors influencing rational drug use among Syrian immigrants in Istanbul. | Cross sectional study | Other/unspecified | Yes | Acceptability, Availability, | Rational antibiotic use practices and knowledge among Syrian immigrants in Istanbul. | - 87.1% of immigrants reported preferring to consult a physician when ill - Self-medication practices were common, and had low adherence to antibiotic regimens, and recommended medications to relatives experiencing similar symptoms |
|  | Dunn-Navarra, 2012, United States | To describe the influence of health literacy on parental knowledge and attitudes/beliefs surrounding upper respiratory tract infection care in an immigrant Latino population. | Cross sectional study | Other/unspecified | No | Appropriateness | Antibiotic classifications and indications | - Latino parents with poorer health literacy were more likely to misclassify certain medications as antibiotics and more likely to incorrectly believe that antibiotics were required for viral infections. |
|  | Eckel, 2014, Germany | To analyze the prevalence and correlations of self-medicated and prescribed drug use for the treatment of common colds (CCs) and upper respiratory tract infections (URTIs) among children and adolescents in Germany. | Cross sectional study | Other/unspecified | Yes | Appropriateness | Antibiotic use among pediatric patients prescribed drugs | - Results suggest a significantly higher likelihood of antibiotics use among children and adolescents of immigrant background compared to non-immigrants. This may be the result of physician prescribing practices, rather than immigrant practices. |
|  | Flaskerud, 1996, United States | To determine how commonly injectable medications were used in the home. | Other: Mixed methods | Other/unspecified | Yes | Approachability, Acceptability, Appropriateness, Affordability | Percentage of users using injectable medications, and how they were used | - At-home medication injections occurred in 43% of respondents’ households and were regular or recurring practice in 14% of households. - Respondents used antibiotic injections because they were more affordable, and they felt that US doctors would not provide adequate treatment. - Needle and syringe reuse was common in nearly half the respondents using injected medications. |
|  | Gammoh, 2016, Jordan | To describe medical complaints and medication consumption in a Syrian refugee population residing in north Jordan. | Cross sectional study | Refugees (unspecified) | Yes | Appropriateness, Approachability | Anti-infectives as proportion of medications dispensed | - Nearly one fifth of prescriptions obtained were anti-infectives. |
|  | Gimeno-Feliu, 2009, Spain | To observe differences in prescription drug utilization between immigrant and Spanish paediatric populations residing in a Spanish region. | Cross sectional study | Labour migrants | Yes | Affordability | Patients' prescription rates | - Prescription drug utilization was lower among immigrant children by 16% when compared to Spanish children. |
|  | Gimeno-Feliu, 2016, Spain, Norway | To compare the relative differences in pharmacological use between natives and immigrants from the same four countries of origin living in Spain and Norway. | Cross sectional study | Other/unspecified | No | Availability, Affordability, Approachability | Proportion of immigrants in Spain and Norway that purchased pharmacological drugs compared to native population | - Immigrants (except Moroccans in Norway) had a significantly lower drug purchasing rate (OR<1 including CI) for antibacterials and penicillins. |
|  | Gonzalez-Lopez, 2012, Spain | To estimate non-prescription pharmaceutical use in the Latin American adult immigrant population of Seville. | Cross sectional study | Other/unspecified | Yes | Appropriateness, Acceptability | Prevalence of self-medication in relation to other variables (age, occupation, time living in Spain, etc.) | - Self-medication among Latin American migrants in Seville is high (77.4% of the sample self-medicated in the past 6 months). The prevalence of self-medication by the type of pharmaceutical was higher with anti-inflammatories, analgesics, and antibiotics. - Migrants that were older, had resided in Spain for a longer time, and had not visited a physician in years were more likely to self-medicate. |
|  | Hedin, 2006, Sweden | To find possible explanations for different antibiotic prescription rates in children. | Cross sectional study | Labour migrants | Yes | Appropriateness | Antibiotic prescription rates in children | - Both parents being born outside of the country is significantly associated with a higher likelihood of being prescribed an antibiotic. - Antibiotic knowledge, concern about infectious disease, infectious symptoms lasting more than 7 days, and physician consultations decreased likelihood of being prescribed an antibiotic. |
|  | Henricson, 1998, Sweden | To investigate the intra-urban variation of antibiotic utilization in children in Malmö. | Other: Ecological analysis | Other/unspecified | No | Appropriateness | Correlation coefficient for dispensation of antibiotics and ethnic status | - There is a negative correlation between ethnic status (being foreign born or a child of foreign-born parents) and antibiotic dispensation generally, across nearly all age groups. - A positive correlation was found for penicillin across all age groups. |
|  | Henricson, 1998, Sweden | To elucidate the relations between demographic and socioeconomic factors, morbidity, and the utilization of major drug groups in an urban Swedish population. | Other: Ecological Analysis | Other/unspecified | Yes | Appropriateness | Prescriptions dispensed | - Antibiotic prescription dispensation was negatively correlated with ethnic status (with a 95% CI which suggests this finding is not statistically significant). |
|  | Hermsen, 2025, Global | To explore and assess global AMR data among refugees and asylum seekers. | Qualitative research | Refugees (unspecified); asylum seeker | Yes | Approachability, Appropriateness | Factors influencing AMR carriage and infections among refugees and asylum seekers | - Poor living and transit conditions increae exposure to AMR. - Access to healthcare in host countries remains a challenge due to cost, language, limited health literacy, unfamiliarity with health systems, differing norms for antibiotic access, low trust, and experiences with discrimination at healthcare facilities. |
|  | Hesari, 2024, Uganda, Yemen, Colombia | To identify key barriers refugees and migrants experience when accessing quality-assured and affordable antimicrobials. | Cross sectional study | Refugees (camp based); migrants (unspecified) | Yes | Acceptability, Availability, Affordability | Barriers to access to quality-assured and affordable anitmicrobials | - Financial, structural, and systemic barriers combined with low AMR awareness and distrust in healthcare providers promote reliance on informal antibiotic markets and self-medication practices. |
|  | Hogenhuis, 2010, Netherlands | To determine whether ethnic origin is an independent determinant for the frequency of antibiotic treatment in primary care patients with infectious diseases. | Cross sectional study | Other/unspecified | Yes | Approachability | Rates of antibiotic prescription | - Findings show that first generation immigrants were significantly more likely to be prescribed antibiotics than the native Dutch population. - No difference in antibiotic prescription was found between second generation immigrants and the native Dutch population. |
|  | Horton, 2012, United States | To determine the role of occupational vulnerability in driving self-medication practices in Mexican migrants working on a farm in California's central valley. | Qualitative research | Labour migrants; Irregular migrants | Yes | Approachability, Acceptability, Availability, Affordability, Appropriateness | Reasons for self-medication and means of acquiring informal medicine | - Respondents acquired medication outside of the formal American health system through multiple means; crossing the border to Mexico to obtain medicines in the pharmacies, sharing with members in their social networks, or acquiring them in corner stores or flea markets. - Occupational vulnerability is a major influence on self-medication practices, followed by cost/lack of insurance, mistrust for the American healthcare system (*e.g.,* being treated poorly, long wait times, etc.). |
|  | Hu, 2015, Australia | To estimate the prevalence of self-medication with antibiotics for treating URTIs and to understand Chinese migrants knowledge of antibiotics and perceptions about use and its association with self-medication with antibiotics for URTIs | Cross sectional study | Other/unspecified | Yes | Approachability, Acceptability, Appropriateness | Knowledge, attitude, and perceptions of antibiotics | - Approximately 20% of surveyed Chinese migrants reported using antibiotics without medical consultation in the past 12 months. - The majority of non-prescribed antibiotic users obtained these antibiotics from leftovers available at home. - A satisfactory experience with a GP service is positively correlated with a decreased likelihood of using non-prescribed antibiotics. - Common barriers to accessing and using GP services include the financial cost of visiting a GP; a lack of trust in GPs; a lack of time for a GP visit, communication issues with the GP; and a lack of transportation for a GP visit. |
|  | Hu, 2016, Australia | To investigate the practice of non-prescribed antibiotic use among Chinese migrants in Australia | Cross sectional study | Other/unspecified | Yes | Approachability, Availability, Affordability | Percentage of antibiotics taken in the last 12 months | - Findings show that foreign born patients were less likely to be prescribed antibiotics or to have consumed antibiotics in the last month compared to the host population |
|  | Imanpour, 2024, United States | To explore structural and cultural factors that lead to antibiotic misuse of migrants who come from countries with easier access to antibiotics. | Qualitiative research | Refugees (unspecified); asylum seekers; labour migrants; migrants | Yes | Acceptability, Affordability | Influences for migrants and refugees using nonprescribed antibiotics | - Economic insecurity, limited insurance coverage, and limited understanding of antibiotics led to bringing antibiotics from their home country and self-medicating |
|  | Jenks, 2016, United States | To assess the prevalence of CA-MRSA/MSSA among patients with SSTIs in CHCs in New York City and the surrounding area, and to identify risk factors and clinical outcomes, including recurrence, of CA-MRSA/MSSA infection. | Cross sectional study | Other/unspecified | No | Appropriateness | Rates of antibiotic prescriptions | - Foreign born patients were less likely to be prescribed antibiotics or have consumed antibiotics in the last month |
|  | Jensen, 2022, Denmark | The study aimed to identify and characterise antibiotic heavy users among elderly patients in general practice with respect to sociodemographic variables. | Cross sectional study | Other/unspecified | Yes | Approachability, Acceptability | Individuals that redeemed an antibiotic prescription from a general practice in 2017 | - Being born in countries outside Scandinavia (Denmark, Norway and Sweden) lowered the odds of being a heavy antibiotic user. |
|  | Kleinert, 2020, Germany | To provide an overview of antibiotic prescription behavior in primary refugee healthcare in Germany. | Cohort study | Refugees (camp based); Asylum seekers | Yes | Appropriateness | Guideline concordance | - Findings indicate that patients often received prescriptions for antibiotics when non-indicated for the diagnosis and received prescriptions for antibiotics that were not aligned with the national guidelines - sometimes it was the wrong antibiotic, and sometimes the patient did not meet all the necessary criteria for prescription. |
|  | Knopf, 2013, Germany | To assess the prevalence rates, determinants, and spectrum of off-label medicine use. | Cross sectional study | Other/unspecified | No | Appropriateness | Prevalence rate of off-label medicine use among those who used medicines | - Off-label use of antibiotics (not disaggregated by migrant background) was most commonly a result of under dosing. |
|  | Ladines-Lim, 2023, United States | To assess refugees’, asylum seekers’, and migrants’ health literacy in appropriate antibiotic use and AMR in the Untied States. | Cross sectional study | Refugees (unspecified); asylum-seekers; migrants (unspecified) | Yes | Appropriateness | Self-reported survey on health literacy and antibiotic use | - In comparison to native-born Americans or those from high-incomec ountries, refugees, asylees, and migrants froms low- and middle-income countries had limited knowledge on antibiotic use and AMR. |
|  | Larson, 2003, United States | To determine self-reported prevalence and correlates of antibiotic use in Hispanic households. | Other: Mixed methods | Labour migrants | Yes | Affordability | Self-reported prevalence and correlates of antibiotic use in Hispanic households | - Being US born is a significant predictor of seeking medical care, but no significant differences between country of birth and antibiotic use was established. |
|  | Larson, 2009, United States | To assess the impact of a culturally appropriate, home-based educational intervention on the knowledge, attitudes, and practices regarding prevention and treatment of URIs among urban Latinos. | Non-randomised experimental study | Other/unspecified | No | Appropriateness | Proportion of participants that believe they should take antibiotics before getting sick | - No significant difference in knowledge scores regarding the treatment of upper respiratory infections were reported between participants of foreign-birth and those born in the US, pre- and post-intervention. |
|  | Laytner, 2023, United States | To explore how acculturation and norms influenced Hispanic indivduals’ use of antibiotics without a prescription. | Cross sectional study | Other/unspecified | Yes | Acceptability, Affordability | Acculturalation levels and norms that lead to inappropriate antibiotic use | - Lower levels of acculturation and social norms supporting non-prescription antibiotic use were linked to greater intention to self-medication with antibiotics |
|  | Lescure, 2022, Netherlands | To establish GPs and pharmacists perceptions, attitudes, and experiences regarding the provision of antibiotics to immigrant patients. | Qualitative research | Labour migrants and other/unspecified | No | Appropriateness | GPs’ and pharmacists’ perceptions, attitudes and experiences regarding the provision of antibiotics to immigrant patients | - Migrants, especially recent ones, may have higher expectations of receiving antibiotics due to familiarity with more liberal prescription practices in their home countries. However, expectations decrease with time or among those accustomed to the system. - Not all migrants engage in inappropriate antibiotic use, but many feel pressured to resolve illnesses quickly due to financial concerns or job insecurity, exacerbated by a lack of sick pay. - GPs may overprescribe antibiotics to maintain patient satisfaction and due to diagnostic uncertainty stemming from language barriers and appointment timing. |
|  | Lindenmeyer, 2016, United Kingdom | To explore the factors that shape migrants experiences of and attitudes to antibiotics. | Qualitative research | Refugees (unspecified), Asylum-seekers, Labour migrants and Other/unspecified | No | Approachability, Appropriateness | Interviews with migrants to understand health beliefs, engagement with health services, transnational medicine, and experiences around antibiotics | - The ease of acquiring antibiotics in a migrant's country of origin explains some of the dissatisfaction with the slow pace and lack of antibiotic prescription from general practioners in the United Kingdom. - The conflicting antibiotic prescription expectations between recent migrants and British GPs can lead to a loss of trust in GPs and push migrants to access antibiotics elsewhere. |
|  | Ljungqvist, 2025, Global | To map socioeconomic factors driving antimicrobial resistance in humans. | Qualitative research | Other/unspecified | Yes | Acceptability | Drivers of antimicrobial resistance | - Self-medication with antibiotics is variable, with studies supporting a correlation between migrant status and antimicrobial resistance; however evidence from Hong Kong highlights that migrants were less likely to self-medicate with antibiotics in comparison to local-born individuals. |
|  | Macias, 2001, United States | To estimate the extent of cross-border utilization of health care services and drug purchases in Mexico among foreign-born Latino adults in the South Bay area of Los Angeles County. | Cross sectional study | Other/unspecified | Yes | Affordability | Percent of respondents that had crossed the border to seek medical care and the reasoning | - Immigrants who crossed the border, most commonly obtained antibiotics. - Cost was a primary barrier to accessing healthcare in the US, especially among the uninsured. |
|  | Madden, 2017, United Kingdom | To understand the health needs and health service experiences of the Eastern European population in a town in Northern England. | Qualitative research | Labour migrants | No | Approachability | Interviews with participants to understand their knowledge of the United Kingdom health service structure, levels of dissatisfaction with GPs and associated justifications | - Migrants experienced dissatisfaction with the health advice obtained from GPs, *e.g.,* GPs overlook health concerns, overprescribe paracetamol, fail to prescribe antibiotics. |
|  | Mainous, 2005, United States | To investigate antimicrobial practices among Latinos living in the United States. | Qualitative research | Labour migrants; Irregular migrants | No | Approachability, Acceptability, Affordability | Proportion of adults in intervention and control community with some exposure to messages about inappropriate antibiotic use | - A substantial portion of respondents purchased antimicrobials outside the US, imported them into the US, and planned to do so again. - Reasons for external purchases include greater comfort with drugs from their home country, to save money, to avoid having to see a doctor, to overcome language barriers, and to have antimicrobials on hand for future illness/to treat someone else. - Respondents used antimicrobials for nonindicated conditions. |
|  | Mainous, 2008, United States | To examine self-medication practices with antibiotics and the acquisition of antibiotics without a prescription among Latino immigrants. | Qualitative research | Other/unspecified | No | Approachability, Acceptability, Affordability | Experiences in treating common infections using self-medication with antibiotics | - Previous diagnoses for similar symptoms from HCWs was a key reason for self-diagnosis and antibiotic self-medication. - Barriers to physician consultation (financial and linguistic) pushed migrants to seek antibiotics without a prescription, most commonly having relatives from abroad mail antibiotics to the US and acquiring antibiotics at *tiendas* (Latino ethnic stores). |
|  | Mainous, 2009, United States | To evaluate a culturally sensitive educational intervention to decrease antibiotic self-medication among Latino communities. | Non-randomised experimental study | Other/unspecified | Yes | Approachability, Acceptability, Affordability | Likelihood to acquire or import antibiotics or believe they should be available. | - Past purchase of antibiotics outside the US is the strongest significant predictor of misuse, including importing antibiotics into the US or acquiring antibiotics. |
|  | McKee, 1999, United States | To assess beliefs, care seeking behaviour, use of antibiotics and means of obtaining antibiotics for the treatment of upper respiratory infections (URIs) among different ethnic groups in an urban community. | Cross sectional study | Other/unspecified | No | Approachability | Likelihood of seeking care, perceived effectiveness of treatment methods, and use of antibiotics not prescribed by physician for a URI | - Respondents who were born or whose mother was born outside the US in a country with over-the-counter antibiotics, were more likely to use non-prescribed antibiotics or to seek out antibiotics for non-indicated reasons. - Barrier to appropriate use among immigrants may be experiences/regulations in their country of birth. |
|  | McMahon, 2007, Republic of Ireland | To compare Irish asylum seekers to other General Medical Scheme patients possessing Irish citizenship in terms of their utilisation of GP services, morbidity patterns and consultation outcomes. | Case control study | Asylum-seekers | No | Appropriateness | Antibiotics as percentage of total prescriptions | - Asylum seekers were more likely than Irish-born citizens to be prescribed antibiotics. |
|  | Moro, 2009, Italy | To investigate determinants of antibiotic prescription in paediatric care in a northern Italian region. | Other: Mixed methods | Other/unspecified | No | Appropriateness | Knowledge, attitude, and practices related to antibiotics | - Paediatricians' attitudes tend to be biased towards antibiotic overuse. - Two principal determinants of overprescribing were identified: diagnostic uncertainty, perceived parental expectations of an antibiotic prescription, and difficulty assuring follow-up. - The variable "parents born abroad"" was significantly associated with a higher odds of receiving an antibiotic prescription. |
|  | Myriam, 2020, Switzerland | To analyse and compare the epidemiology and spectrum of diseases in asylum-seeking and non-asylum-seeking children. | Cohort study | Other/unspecified | No | Approachability | Accounting-units per admission | - Amoxicillin-clavulanic acid and cephalosporins were the most commonly prescribed antibiotics overall. |
|  | Nanakali, 2023, United Kingdom | To explore antibiotic use of migrants living in the United Kingdom. | Qualitative research | Migrants (unspecified); refugees (unspecified); asylum-seekers | Yes | Acceptability, Availability, Affordability,  Approachability | Interviews with participants to understand living conditions and wider factors contributing to their antibiotic use. | - Migrants often brought antibiotics from their home countries because of unfamiliarity with the UK health system, long wait times, and costs of medicine - Language barriers and mistrust of health care providers also led to self-medication practices |
|  | Ozcebe, 2022, Turkey, Germany, Sweden, Netherlands | To explore attitudes and behaviors of physicians and pharmacists regarding prescription or supply of antibiotics as well as their experiences and perspectives about rational antibiotic use among adults in Turkey and Turkish migrants in Germany, Sweden and the Netherlands. | Qualitative research | Other/unspecified | No | Approachability, Appropriateness | Attitudes and behaviours of physicians and pharmacists towards antibiotic use amongst Turkish migrant and non-migrant adults. | - Turkish migrants, particularly younger individuals, trust their physicians but practice self-medication, especially migrants in Germany. - Frustration with differences in healthcare systems and language barriers in their host countries, and ease of access to antibiotics in Turkey, may drive Turkish migrants to self-medicate. |
|  | Panagakou, 2012, Greece | Identify risk factors associated with antibiotic misuse in Greece. | Cross sectional study | Refugees (unspecified) | Yes | Appropriateness | Knowledge, attitudes and practices on antibiotic use | - Questionnaires revealed that being an immigrant was a statistically significant risk factor for not answering the KAP sections on antibiotic use correctly compared to non-immigrants. However, other factors such as being a father or having a low educational level played just as significant a role in low KAP for antibiotic use within the population. |
|  | Piegeolet, 2018, Jordan, Lebanon, Gaza Strip, West Bank | To assess the differences in medical officers’ knowledge and beliefs about antibiotics. | Cross sectional study | Refugees (camp based) | No | Appropriateness | Knowledge of AMR | - High prescription (prescribing antibiotics for over 25% of URTI cases) was associated with different variables for the different groups - clinical experience and patient's parents' education level |
|  | Polonsky, 2021, Bangladesh | To document the epidemiological, clinical, and public health response characteristics of a large outbreak of diphtheria among Rohingya and the local host population in Cox Bazar district. | Descriptive/ observational | Labour migrants, irregular migrants, other/unspecified | No | Appropriateness, Availability | Proportion of patients receiving antibiotics | - Of the over 7000 cases of diphtheria (including lab-confirmed, probable and clinically suspected cases), only 62% received antibiotics. - Contact tracing was performed for a majority of cases, and most contacts were given prophylactic antibiotic treatment. |
|  | Pylypa, 2001, United States | To describe and compare self-medication practices in two California Mexican immigrant groups: families living along the border near Tijuana, Mexico, and migrant farm worker families residing in illegal encampments and sub-standard housing in San Diego's North County. | Qualitative research | Other/unspecified | Yes | Approachability, Acceptability, Availability, Affordability | Interviews on information related to health-seeking and decision-making, barriers to formal health services use, self-treatment practices including injection use, and treatment of recent illnesses in the family | - Informants were more likely to purchase and self-medicate with antimicrobials from Mexico because of lack of financial resources, language barriers, low education rates, social norms, dissatisfaction with US physician treatments, and easy access to Tijuana. |
|  | Rahill, 2012, United States | To identify the type and frequency of substances injected in a sample of adult Haitian immigrants residing in Miami-Dade County, Florida. | Case control study | Other/unspecified | Yes | Approachability, Acceptability, Appropriateness, Affordability | Antibiotic use among Haitian immigrants | - 70% of immigrants reported routine injections of broad-spectrum antibiotics such as penicillin, ampicillin, tetracyline, and streptomycin. |
|  | Rasmussen, 2021, Denmark | To compare knowledge of appropriate use of antibiotics and antibiotic resistance among native and foreign healthcare workers in Danish nursing homes. | Non-randomised experimental study | Labour migrants | No | Appropriateness | Knowledge of antibiotics | - Overall, native HCWs had a higher knowledge of antibiotic than foreign HCWs. even after adjusting for work experience, place of employment, and educational background. |
|  | Sahlan, 2008, Germany | Evaluation of a German leaflet adaptation designed to improve doctor-patient communication for Turkish patients. | Qualitative research | Refugees (non-camp based) | No | Approachability | Participant understanding of leaflet messages and changes in their attitudes after reading the leaflet | - Patients have different views on the level of engagement they should have with their doctors in consultations when it comes to antibiotic prescription, with female patients gender seeking to be more involved. |
|  | Saif, 2022, United States | To estimate the prevalence and success of the treatment of H.pylori in refugee adult and pediatric patients attending a refugee primary care clinic, and to identify barriers to treatment. | Cross sectional study | Other/unspecified | Yes | Acceptability | Interviews with Turkish patients | - Those who tested positive for H. Pylori infections were often prescribed antibiotics. - Challenges to H. pylori care for refugees included: Ramadan (fasting), infection spreading within families, pregnancy and language/cultural barriers |
|  | Sanchez, 2014, United States | To expand knowledge of self-medication practices among Latino immigrants in the US. | Other: mixed methods | Other/unspecified | No | Approachability, Acceptability, Appropriateness, Affordability | Treatment outcome | - Lack of comfort in their current health system compared to their country of origin, high cost of the US health system, a lack of insurance, a lack of transportation, antimicrobials being perceived as stronger and coming with Spanish instructions, and fear of deportation were cited as barriers to accessing healthcare in the US, and resulting in a preference for drugs from their countries. |
|  | Sapin, 2025, Canada | To examine how social determinants of healthcare access influenced antibiotic prescribing before and after COVID-19. | Cohort study | Other/ unspecified | Yes | Appropriateness | Incident of outpatient antibiotic prescriptions | - Antibiotic prescribing was higher among recent immigrants compared to long-term residents. - The difference was more pronounced during the COVID-19 pandemic (sHR, 1.21 [1.18–1.25]) than pre-pandemic (sHR, 1.12 [1.09–1.16]). |
|  | Schuts, 2019, Germany | To determine whether appropriate knowledge and use of antibiotics differ by ethnicity and whether knowledge on antibiotics is associated with antibiotic use. | Cross sectional study | Other/unspecified | Yes | Appropriateness | Knowledge of antibiotics, and antibiotic use | - Compared to Dutch individuals, first- and second-generation Ghanaian individuals and first-generation Moroccan individuals had significantly higher odds of receiving an antibiotic prescription. - Second generation migrants and females generally associated with higher levels of antibiotic knowledge. |
|  | Shutt, 2025, Global | To review knowledge, attitudes, and practices of AMR among socially vulnerable and marginalized populations, and how these relate to social determinants of health. | Qualitative research | Migrants (unspecified); refugees (unspecified); asylum-seekers | Yes | Acceptability | Knowledge, attitudes, and practices of AMR | - Migrants, refugees, and asylum seekers show conflicting attitudes toward doctor’s role in antibiotic prescriptions, with some expecting them regardless of medical advice and others following doctors’ decisions. - Previous successful antibiotic use often led to repeat self-medication without consulting healthcare providers. - Self-medication was common and persisted among migrants, refugees, and asylum seekers due to healthcare access barriers and dissatisfaction with healthcare services in host countries. |
|  | Shehnaz, 2014, United Arab Emirates | To investigate the knowledge, attitudes, of medicines and self-medication among expatriate adolescents in the United Arab Emirates. | Cross sectional study | Refugees (non-camp based) | Yes | Appropriateness | Correctly identifying the definition of an antibiotic, their necessity for cure, and completing the prescribed course | - A total of 289 from 324 students reported to be self-medicating. - The mean total drug knowledge scores of those who self-medication and those who do not were 12.11 ± 4.32 and 12.03 ± 4.53, respectively. There was no significant difference between the two groups. |
|  | Silva, 2025, United Kingdom | To investigate migrants’ experiences with antibiotic use and healthcare access in the UK, and how this may affect AMR risk. | Qualitative research | Migrants (unspecified); refugees (unspecified); asylum-seekers | Yes | Approachability, Acceptability | Interviews with UK migrants about their experiences with antibiotic use and healthcare access | - Migrants faced difficulties accessing GP appointments, leading to delays in care and increased self-medication using leftover or imported antibiotics. - Many stored antibiotics at home for “emergencies,” reflecting mistrust in access to timely healthcare and a desire for control over treatment. - Incomplete antibiotic courses and repeated use for similar or persistent symptoms created a self-perpetuating cycle of misuse. Knowledge of “red-flag” symptoms and how to communicate with GPs was used strategically to obtain antibiotics. - Experiences with healthcare providers were mixed—trust improved with interpreters and cultural sensitivity, but barriers like language, cultural expectations, and lack of face-to-face care fueled self-treatment. |
|  | Simonek, 2023, Greece | To investigates the prevalence of common infections and patterns of antibiotic use at a healthcare facility within Moria refugee camp, Lesvos, Greece, during the winter and summer seasons of 2019. | Other: Mixed methods | Refugees (camp based); asylum-seekers | Yes | Appropriateness | Prevalence and seasonal variation of common infections and antibiotic prescribing patterns in Moria refugee camp, Greece | - Inequalities in access to the clinic exist, particularly for unaccompanied minors, women, children, and people with disabilities. - Antibiotic prescribing was influenced by limited diagnostic resources, leading clinicians to rely on clinical judgment and prescribe cautiously due to overcrowded, unhygienic conditions and high patient susceptibility, with prescribing patterns also varying by clinician training background. |
|  | Spjeldnæs, 2025, Lebanon | To explore how residents of Shatila refugee camp in Lebanon access and use antibiotics within the constraints of poverty and limited healthcare, and how their coping strategies can inform locally driven interventions to reduce antibiotic use. | Descriptive/ observational | Refugees (camp based) | No | Availability, Accommodation, Appropriateness, Affordability | Interviews and observations on how Shatila residents access and use antibiotics through daily coping tactics. | - Shatila residents relied on informal networks and pharmacies to access antibiotics, often bypassing formal healthcare due to poverty, discrimination, and limited services. - Social networks and personal ties with pharmacy workers or through social media were crucial for accessing medicines affordably. - Residents often self-diagnosed and self-treated, experimenting through trial and error and maintaining “home pharmacies” with leftover antibiotics. - Shatila’s flourishing informal healthcare market provides readily accessible, inexpensive antibiotics, making them the most commonly used form of care. - Despite awareness of overuse risks, residents continued self-medication out of necessity, illustrating coping, creativity, and resilience in navigating constrained healthcare environments. |
|  | Spjeldnæs et al., 2025, Lebanon | To explore the factors influencing antibiotic use within the Shatila refugee camp in Lebanon. | Qualitative research | Refugees (camp based) | No | Acceptability, Affordability, Availability | Interviews with Shatila camp residents on their antibiotic use. | - Antibiotics were easily accessible and used to treat a wide range of ailments, leading most households to keep home pharmacies, though they often failed to relieve illness. - Limited financial means discouraged residents from seeking medical advice, as antibiotics were among the cheapest medicines available. - Weak pharmacy regulation and the presence of informal vendors further fueled the circulation and widespread consumption of low-cost antibiotics. |
|  | Tahir, 2021, Malaysia | To study the types and determine the factors associated with infectious diseases, and the appropriateness of the type of antimicrobials prescribed in Rohingya pediatric patients attending the IMARET mobile clinic. | Cross sectional study | Refugees (camp based) | No | Availability, Appropriateness, Affordability | Percentage of prescriptions with preferred antimicrobial agents. | - Around 18% of patients received an inappropriate antimicrobial prescription for their diagnosis. Limited medication and medical availability, patient records, and knowledge of volunteers, contributed to inappropriate prescription practices. |
|  | Thin, 2025, Global | To systematically examine the prevalence, sources, and factors influencing self-medication among immigrants, as well as the extent of inappropriate self-medication practices. | Qualitative research | Other/ unspecified | yes | Approachability,  Acceptability, Affordability, Appropriateness | Prevalence, sources, and determinants of self-medication among immigrants | - Fear of deportation, high healthcare costs, lack of insurance, language barriers, and limited counseling from healthcare providers drove self-medication. - Immigrants obtained medicines from home countries, family/friends, local markets, and informal networks. - Prevalence varied by host country, origin, culture, and healthcare system, with immigrants in Thailand and Norway 4–6 times, and in Spain 1.3 times, more likely to self-medicate than host populations. |
|  | Tresachez-Lacorte, 2020, Algeria | To describe the medicines found a sample of Sahrawi homes in one district of the refugee camp in Tindouf, with special focus on antimicrobials. | Cross sectional study | Labour migrants | No | Acceptability | Antimicrobials found in households | - Antimicrobials made up 15.6% (42/269) of all the medicines found, in addition to being present in more than half of the 76 surveyed households (42/76). - The most common antimicrobials found were amoxicillin, a combination of amoxicillin and clavulanic acid, and metronidazole. - The majority of the antimicrobials found in the refugee households had been obtained via prescription (83.3%) and had a valid expiration date (95.2%). - Families that had antimicrobials at home had used them for non-infectious diseases in more than one third of cases (36%), and that the dosage and duration of treatment for the antimicrobials were inappropriate (underused) in almost half of the cases (43%). |
|  | Väänänen, 2006, Spain | To determine the frequency of self-medication of antibiotics among Finnish adult population living in southern Spain. | Cross sectional study | Labour migrants | Yes | Approachability, Appropriateness | Use of antibiotics during the previous 6 months, number of courses taken, and adverse reactions | - Almost half (41%) of surveyed Finns that had recently used antibiotics had obtained some or all of them without a prescription. Factors such as age, gender, marital status, and working situation did not affect the likelihood of non-prescription antibiotic use among Finns living in Spain. |
|  | Vissman, 2011, United States | To explore access to and use of prescription drugs obtained from local nonmedical sources among immigrant Latinos in the southeastern US. | Qualitative research | Other/unspecified | Yes | Approachability, Acceptability, Appropriateness | Interviews with Latino community members to gain perspectives on use of nonmedical sources of prescription drugs | - Participants revealed alternative ways of acquiring medications outside of medical settings, mainly through *tiendas* (Latino ethnic stores) as well as by having relatives mail medications to them or bring them across the border. - Participants cited the 'over-regulation' of prescription medicines in the US as a reason for resorting to self-medication. - Cultural norms and stigma around STIs may push certain Latino migrants (especially males) to seek medicines from non-medical sources. |
|  | Wemrell, 2022, Sweden | This study aimed to provide a more precise mapping of how the dispensation of antibiotics is distributed across socio-economic and demographic groups in Sweden. | Cross sectional study | Other/unspecified | Yes | Appropriateness | Prevalence ratios of antibiotic dispensation | - While dispensation was more common in older age groups and among women, no substantial differences pertained to country of birth. |
|  | Westerling, 2020, Germany, Netherlands, Sweden & Turkey | To explore the variation in implemented policies related to AMR in Germany, the Netherlands, Sweden and Turkey and the perceived access to antibiotics and information on rational antibiotic use among Turkish migrants living in the three EU countries and citizens in Turkey. | Qualitative research | Other/unspecified | No | Approachability, Appropriateness | Interviews to understand variation in implemented policies related to proper antibiotic use in Turkish citizens | - Few migrants obtained antibiotics without a prescription and had similar attitudes and expectations on antibiotic prescription as the host population. - While most of the Turkish migrants reported taking antibiotics only when prescribed and as instructed, some respondents reported that alternative ways of acquiring non-prescribed antibiotics were available to them (travel to Turkey, online access, leftovers from family members). - Respondents highlighted that the lack of verbal and written communication from physicians and pharmacies (especially in their native language) and trust in physicians was a main barrier to understanding the broader concept of rational use of antibiotics. |
|  | Whittaker, 2019, Australia | To describe the understanding and experiences related to antibiotic use and AMR of people from diverse ethnic backgrounds in Australia. | Qualitative research | Refugees (unspecified); other/unspecified | Yes | Approachability, Acceptability | Interviews | - Differences in how antibiotics are perceived in one's country of origin can play a role in migrant's avoidance or misuse of antibiotics, where informants had little to no knowledge of AMR. - Migrants and interpreters reported bringing back prescribed or purchased antibiotics from their countries as they were easier to obtain, however one individual also reported fear of getting low quality/counterfeit medications. - Migrant mothers preferred traditional medical practices from their countries before resorting to antibiotic use. |
|  | Wolcott-MacCausland, 2020, United States | To understand what factors influence the decision of Latina dairy workers (and household members) living in Vermont to request health products from Mexico by mail. | Other: Mixed methods | Labour migrants | No | Approachability, Acceptability, Availability | Interviews to understand the factors that contribute to the use of mail-ordered medication among Latina migrant dairy workers | - Half of the participants (17/34) stated that they had requested and used health products (including antibiotics) sent by mail from their countries of origin in the past year. - Proximity to the international border - whether directly or indirectly - played a role to request medications by mail. - Although Latina dairy workers could request medicine from their employer or the health promoters, they often would not be the used due to factors such as the employer's limited time or lack of an established relationship with health promoters. Other commonly cited barriers included language barriers, a lack of knowledge on available health care options, and a lack of transportation, and time due to wok. |
|  | Yusuff, 2023, Global | To describe the rising forcibly displaced persons (FDP) population, the factors contributing to their AMR burden, and existing surveillance gaps. | Qualitative research | Migrants (unspecified); refugees (unspecified); asylum-seekers | No | Availability | AMR among FDPs | - Poor living conditions and limited access to clean water, sanitation and healthcare among FDPs contribute to the spread of AMR. |
