# Supplementary material for: A rapid scoping review of antibiotic access and use barriers among refugee and migrant populations
Source: Global Health. 2026 Jan 29;22:20. doi: 10.1186/s12992-026-01188-x (PMC12874966; doi:10.1186/s12992-026-01188-x)
Supplement: Supplementary file 4 — Supplementary Material 4: Appendix Fig. 1. Prisma chart. [file 12992_2026_1188_MOESM4_ESM.docx]

Studies from databases/registers **(n = 6498)**

Web of Science (n = 1120)

Scopus (n = 466)

MEDLINE (n = 354)

Unspecified (n = 4558)

**Identification**

Studies excluded **(n = 3760)**

Titles and abstracts screened **(n = 4440)**

References removed **(n = 2058)**

Duplicates identified manually (n = 4)

Duplicates identified by Covidence (n = 2054)

Marked as ineligible by automation tools (n = 0)

Other reasons (n = )

Studies excluded **(n = 593)**

Language (n = 15)

TB/HIV/Malaria (n = 15)

Wrong outcomes (n = 259)

Wrong comparator (n = 8)

Wrong intervention (n = 54)

Wrong study design (n = 171)

Wrong patient population (n = 71)

Full-text studies assessed for eligibility **(n = 680)**

**Screening**

Supplementary articles **(n = 38)**

Snowball searches (n=18)

Grey literature searches and expert consultation (n=20)

Studies assessed for eligibility **(n = 83)**

**Included**

Studies included in review **(n = 121)**
